# Exploring the role of microfinance in women’s empowerment and entrepreneurial development: a qualitative study

**DOI:** 10.1186/s43093-022-00172-2

**Published:** 2022-12-08

**Authors:** Ambreen Khursheed

**Affiliations:** UCP Business School, Faculty of Management Studies, University of Central Punjab, 1-Khayaban-e-Jinnah Road, Johar Town, Lahore, Pakistan

**Keywords:** Microfinance, Pakistan, Women empowerment, Women entrepreneurship, I32, O12, O15

## Abstract

In developing countries, women’s empowerment is a major concern. Several efforts were made to tackle this issue as the aims of poverty reduction and development cannot be achieved without giving attention to women’s empowerment. Over the past decades, microfinance institutions (MFIs) have appeared as crucial tools not only to address the issue of poverty but also particularly to empower women. Resultantly, a huge number of studies focus on the relationships between MFI and women empowerment. However, in the context of rural areas of Pakistan, the research is limited. Therefore, the objective of this study is to investigate the role of MFI in women’s empowerment in Pakistan so that the research will facilitate MFIs and policymakers in strengthening the link between MFIs and women entrepreneurship. We have used a qualitative methodology, using primary data collected through in-depth interviews and a focus group discussion with six female borrowers of Rural Community Development Programs (RCDP). The empirical results provide valuable insights into the efforts made by RCDP to empower women and combat poverty by encouraging women’s entrepreneurship. Hence, this paper not only examines empowerment, which women are attaining from microfinance but also assists MFIs to know about their significance in developing the economy. The paper is significant for MFI practitioners to develop policies for boosting women’s entrepreneurship and to help their existing women clients with efficient training and supervision.

## Introduction

Microfinance has a unique ideological demand as compared to charity. It is particularly designed to support poor people. However, it is a long-term process that enables the poor to improve their living standards in an effective manner [39, 41, 74]. In particular, when we talk about microfinance from the perspective of women, the role of benefactors of microfinance seems important in making it a relatively effective resource for poverty alleviation, the stability of economic growth, and women empowerment [25, 39, 41].

The difference between male and female ratios is not considered significant, but in several areas, women are provided less importance and power in comparison with men [29, 37]. Women around the world have little control over their assets and have less political power. Further, they do not have a lot of properties to their name [58, 68, 87]. Due to a lack of security saved in the financial sector, women faced several difficulties during the financial crisis period which lasted from 2007 to 2008 [52]. Similarly, it is crucial to understand the impact of the recent crisis of COVID-19 which affected all businesses badly and also threatened world health security [81].

However, several researchers have questioned this statement. The classification of all expected benefits and disadvantages of MFIs is still in the initial phase. We are still discovering how to improve the living standards of poor women and their families. This study aims to broaden existing knowledge about the role of MFIs in empowering women in rural Pakistan.

In emerging economies, MFIs and women empowerment is considered to be one of the most effective tools for poverty alleviation by particularly focusing on women [62, 87]. Certainly, women are one of the most important parts of society and without their presence, societies cannot improve [23]. Women empowerment leads to the increased participation of females in the workforce, the capability to decide, and poverty reduction. Thus, an increase in their income will not only prove beneficial for their family but will also have a very positive influence on the economy [58]. Another study investigated the nonlinear effect of the education level on the ecological footprint by incorporating the variation in the population and income structures and recommended crucial policies regarding education levels and environmental sustainability [82].

In developing countries, all businesses are male-dominated and females have to suffer from discrimination in most of the phases whether it is their personal life or professional life. However, financial segregation seems complicated for developed nations regardless of the gender factor. Financial stability is a key concern for developing as well as economically challenged countries as these economies do not have a stable financial environment and well-established institutions [42, 43]. The presence of poor health facilities, underdeveloped financial industries, illiteracy, and weak infrastructure have raised serious problems for developing nations. To consider the requirements of financially excluded women, MFIs step forward to help those women in establishing new endeavors [55]. As a result, non-government organizations and government agencies decided to provide subsidized loans for a better lifestyle of people and poverty alleviation. Prior researchers appreciated the initiative of such investments (for example, [25, 60]), but disproportion has been observed in these investments from the side of rich landlords or agencies. To tackle this issue, some highly effective alternative social networks, social collaterals, and credit scoring are needed here to approach the poorest women [57]. Moreover, women in more rigid cultural settings are likely to face a higher risk of domestic violence because economic empowerment intervenes with patriarchy and expedites change in rigidly defined gender roles [27, 28]. Therefore, the need to address gender power imbalance and existing gender roles need to be taken into account before making interventions to empower women. It is found that the main body of the related existing literature primarily discussed only those factors that played a key role in the supply side of agriculture finance and microloans. A few past studies have also focused on the demand side of microfinance loans. However, the study of Guirkinger and Boucher [21] and the study of Ashraf and Ali [7] have highlighted the possible hurdles of the demand side of microfinance loans faced by smallholders. These obstacles include complex application procedures and complexity in providing loan securities. The seminal work of Garikipati et al. [18] reveals that the process of providing loans to the poor is uncertain, and is not easily generalized. So, people should be careful to utilize this development tool. However, it is clear that these loans provide financial benefits for poor women in developing new endeavors [71] and also act as a smart policy to help the poor [10]. Irrespective of the talk of “gender neutrality,” MFI clients that are women of immobile poor backgrounds have a lower default record as compared to men. MFI start-ups usually have significant and underreported economic effects because the poor women who work within households are not getting the standard pay and have limited start-up funds.

Brière and Szafarz [13] reported that MFIs have now become a risk-averse thing and it is “financialized,” i.e., MFIs now act as mainstream financial institutions. On the other hand, MFIs are considered a good source of financial support for women in starting new businesses and a tool to eliminate poverty in the country but this fact is not applicable universally because MFIs can also appear as an enigma in providing microfinance access to women. In various literature studies, researchers have focused on savings and credit products MFIs. It has been found that research studies are showing great interest in microfinance. Therefore, we aim to explore how MFI can lead to women’s empowerment and entrepreneurship. Furthermore, we also decided to investigate the possible benefits of microfinance for women from RCDP’s microcredit program.

### Problem statement

One of the objectives of microfinance is to enhance women’s empowerment and to generate employment opportunities by promoting self-employment that consequently improves the social well-being of poor people. Most of the existing studies, mainly in economics, have only focused on how MFIs lending helps in poverty alleviation, rather than analyzing its impact on social and financial empowerment and new venture creation by women. The majority of the past studies were quantitative [9, 15, 17], while there were a few qualitative studies applied in various contexts that analyzed the impact of MFIs in enhancing women’s empowerment but still substantial studies are not available which explores specific lived experiences of women borrowers when they avail microloans and how they utilize that loan in starting their businesses. Therefore, this study aims to enhance the understanding of the role of microfinance from the viewpoint of beneficiaries in improving their empowerment and entrepreneurial development.

### Significance of the study

Pakistan is a developing country and the majority of its population is living under the poverty line and are mostly unaware of different sources of financial facilities. MFIs particularly focus on such rural areas in which most of the people are un-bankable and marginalized. This study contributes to the extant literature, as it explores the lived experiences of women borrowers regarding empowerment and entrepreneurial development. To get deeper insights into the structural meaning of empowerment analyzed by considering participants’ histories, lived experiences, and social interactions, we used a qualitative approach that relies on in-depth interviews and a focus group under the case study research design. This study provides valuable insights into how MFIs are making women socially and financially empowered. Also, how microfinance helps in women-led ventures’ creation process. To investigate how microfinance is increasing women’s empowerment, we deduced the following sub-objectives.

- To explore how women become socially empowered after getting micro-financed.
- To figure out how women become financially empowered after getting micro-financed.
- To determine how microfinance increases women’s entrepreneurship.

## Literature review

### Microfinance

Microfinance programs have been playing a dominant role in poverty alleviation since long ago [40]. The vision behind the growth of microfinance is to pull the poor toward the entrepreneur side by giving them enough credit to achieve this goal. However, microfinance usually considers one assumption, i.e., the beneficiaries have adequate social capital, human capital, and other required assets for expanding their small-scale businesses. This indicates that the lack of credit is the only prominent hurdle experienced by poor women [73]. This assumption seems quite complicated because the growth of even a small business requires a lot of competencies, knowledge, expertise, and abilities [2]. Another major issue is that microfinance faces difficulty to approach the right poor people [16]. In the light of practical aspects, microfinance refuses the poorest division of people from borrowing money. This violates its role in approaching very poor applicants [14, 83]. Furthermore, the poorest household people who are availing the benefits of microfinance still lack the proper technical skills that are necessarily required for business. The background of microfinance shows it is an essential tool to alleviate poverty, it works by receiving donations and lending money to poor people. Microfinance programs disregard the non-income parameters of poverty such as health, security, and education [11]. The study of Shaw [64] explains how the poorest households possess limited formal education. Also, poor health and undernutrition play a vital role in limiting the overall productivity of such households. The lack of education results in severe illiteracy which can badly affect the poor and make them unable to properly understand the effective working procedure of loans. Famous examples include Akhuwat, AGAHE Pakistan, AMRDO Foundation, non-bank microfinance companies, and many more.

### Measuring empowerment

The study of Malhotra et al. [47] reports that the identification of empowerment as a primary development tool has been done, but still, institutions such as the World Bank and development agencies haven’t introduced an authentic method for estimating and analyzing the tracking variations in various levels of empowerment. Researchers define empowerment as a dynamic procedure that is complex to measure. The reason behind this is that empowerment is related to social, economic, and political challenges as well [63]. The spiritual, social, political, and health factors make the complete empowerment measurement procedure and these all factors are interconnected with each other. The term empowerment can also be expressed as a way of independent decision making, identification, and utilization of resources [1]. The literature reveals that empowerment is a multidimensional concept and it can be assessed under multiple dimensions [31]. This study primarily focuses on the influential impact of microfinance on women’s empowerment in the context of the financial and social aspects. This is because the financial and social aspects of women’s empowerment help increase the development of both the quality and quantity of existing human resources. These two aspects are proven as critical factors in enhancing the development of a society.

### Meaning of women’s empowerment

There is significant diversity in the agendas, emphases, and terminologies used for describing women’s empowerment. Many papers have defined empowerment and its measurement approaches. The most common terms used in the extant diverse approaches use power, choice, control, and the option to describe women’s empowerment [72, 78]. However, it is still confusing to say whether the terms “empowerment”,” “gender equality,” “women’s autonomy,” and “women’s status in society” are similar or different concepts. The term women empowerment has been conceptualized mostly as an outcome or a capacity or some means to an end, and a process of achieving power [35, 54].

### Microfinance and women’s empowerment

Women are the main target audience of microfinance programs. This credit amount not only helps poor women to grow economically but also improves gender equality, the status of women within the family, their health, and their education level [35]. Moreover, women are examined as a good credit risk by microfinance programs due to their increased propensity to repay loans [24]. In contrast, men are more interested in moving their money toward risky business practices and are at high risk to consume this money on tobacco, gambling, or drinking [20]. However, Goetz and Gupta [20] also highlighted that a significant percentage of women’s loans are directly invested in business activities by their male relatives, but the liability of repayment goes to women borrowers. The recent literature primarily discusses the evaluation process of microfinance programs [3, 38, 65] in the context of the well-being of borrowers [14, 50] and empowerment capabilities of women [61]. The reporting of these evaluations reveals some conflicting conclusions, and it still tells that borrowers have an absence of accounts for themselves and this impact of credit can affect their lives [35]. There is limited evidence in the literature on how the poor perceive the process of microfinance loans. In addition, the existing literature has limited scope regarding the “transformative process” of entrepreneurship which reveals the lives of those needy people who are living in extreme poverty [76]. In response, this study fills the gap in the literature by examining how most disadvantaged borrowers or potential borrowers themselves perceive and experience microfinance in a context characterized by extreme poverty, one where family responsibility and entrepreneurial activities are closely intertwined.

A study reported that 95% of Grameen’s borrowers were females and this percentage kept on raising till 2011. Similarly, Aghion and Morduch [6] highlighted that 71% of total borrowers of MFIs were women. Further, past researchers have also pointed out that MFIs target women because their default rates are very low as compared to men [5, 36]. Because of this reason, MFIs have launched several innovative schemes to financially support their female clients. MFIs play a crucial role in enhancing the empowerment of women as it boosts their resources, increase return on human capital by improving their affordability, and consequently improve their living standards.

### Social empowerment of women

Women’s social empowerment refers to having a supportive environment by using different affirmative programs and policies for the empowerment of women along with the provision of easy and equal access to necessities of life [48]. In the field of development, empowerment has become a catchword, with a specific focus on poverty alleviation and the political addition of marginalized groups of women [49]. Microfinance has proved socially beneficial for women [35]. In a pivotal study, Mahmud [46] described that microfinance institutions have a significant positive influence on women’s social empowerment as it substantially improves their control of income spending and intra-household decision-making power, which resultantly enhances their welfare. Sinha et al. [67] found that women’s participation in MFIs enhanced their capability to spend money, mobility, and dominance in household decision making. Further, Montgomery and Weiss [51] concluded women’s participation in MFIs leads to enhance family decision making and found that family landholdings, media exposure, and institutional access are key determinants of women empowerment [26]. Similarly, it was found that savings impact is more significant on women as compared to men as it enhances their decision-making power related to family planning, family expenses, recreation, and their lifestyle [8].

Therefore, there is a need for an integrated microfinance program comprising education with skill-building training for increasing the capacity building of women and fortifying the relationship between women’s social empowerment and microfinance [4].

### Financial empowerment of women

Many past studies have analyzed women’s empowerment from different perspectives; however, financial empowerment is ignored to some extent. In this study, one of the main objectives is to examine the financial empowerment of women. Past studies have reported that financial empowerment can be understood through three factors; financial literacy, financial attitude, and financial well-being. Financial literacy is inherent in humans and is recognized as the primary privilege of humans. “Financial literacy is the capability of understanding finance” [75]. Lack of financial knowledge ultimately pulls poor people away from success in financial markets or businesses [79, 86]. The importance of financial literacy is equal for men and women. However, it is reported that if women have stronger financial knowledge then they can do effective future planning [45]. Financial knowledge is related to financial attitude. The financial attitude refers to the capability to manage finances, interest in enhancing financial knowledge, and investment decisions. Past studies revealed that financial knowledge, financial attitude, and financial behavior affect financial empowerment or financial well-being [33, 66]. The concept of financial well-being is related to personal traits, knowledge of finance, and attitude. Therefore, the subjective meaning of financial well-being varies from person to person [32]. Thus, the financial empowerment of women can be assessed by considering financial literacy, financial attitude, and financial well-being.

## Research gap

The literature discussed following the structure from the history of microfinance to concepts of women empowerment leads to the discussion on the relationship between women empowerment and microfinance. The literature depicts that different indices were explained in prior research studies giving a quick overview of empowerment but they are limited as they used a few variables, ignored key ontological issues, details, and subjective experiences that deepen the understanding of empowerment [9, 15, 17]. Therefore, this study fills the existing gap as we interviewed women in their natural settings and in their contexts in which they interpreted empowerment from their viewpoints.

Further, there was a strong practical gap regarding the lack of research on how women experienced empowerment and entrepreneurship through microfinance. A majority of the past studies applied quantitative methodology with the top-down approach which focuses on the views of service providers instead of beneficiaries and thus the beneficiaries’ views were not considered. Therefore, it becomes evident that the quantitative approach is not suitable for understanding women’s empowerment because it is a process of realization and only participants can explain what empowerment means to them through their experiences and feelings of becoming empowered. Hence, it is significant to use a qualitative methodology to capture the real feelings and experiences of women. Therefore, we applied the bottom-to-top approach to analyzing the true essence of the lived experiences of women regarding empowerment and entrepreneurship. Thus, this study is based on a case study research design to explore the perspectives of women that how they interpret and understand the phenomenon of empowerment achieved through microfinance in their natural context. Overall, this study enriches the extant literature about women’s empowerment by explicating the complex phenomenon of empowerment through social, financial, and entrepreneurial contexts.

## Research question

For exploring the effectiveness of MFIs in terms of women’s empowerment and entrepreneurial development, we propose the main research question of this study as follows;

- What is the impact of microfinance on women’s empowerment?

**Sub-questions**

- How does a woman become socially empowered after getting microfinance?
- How does a woman become financially empowered after getting microfinance?
- To what extent microfinance leads to women’s entrepreneurial development?

## Theoretical framework

### William’s theoretical model of women’s empowerment

In this study, two theories as theoretical frameworks are used. The first theory is by Williams [84] who formed a theoretical model on women’s empowerment. In the development of this model, the innovative insights of Kabeer [34] were used. Given this theory, empowerment comprises three factors, resources, agency, and achievements. Here, the resources present the supporting factors which are utilized by women to achieve empowerment, the agency presents the ability of the women to achieve their goals, and achievement refers to the success of women in achieving their life goals. Resultantly, the results achieved represent achievements by combining resources with the agency. We have used this model for measuring women’s empowerment.

### Status withdrawal theory

**Fig. 1:**
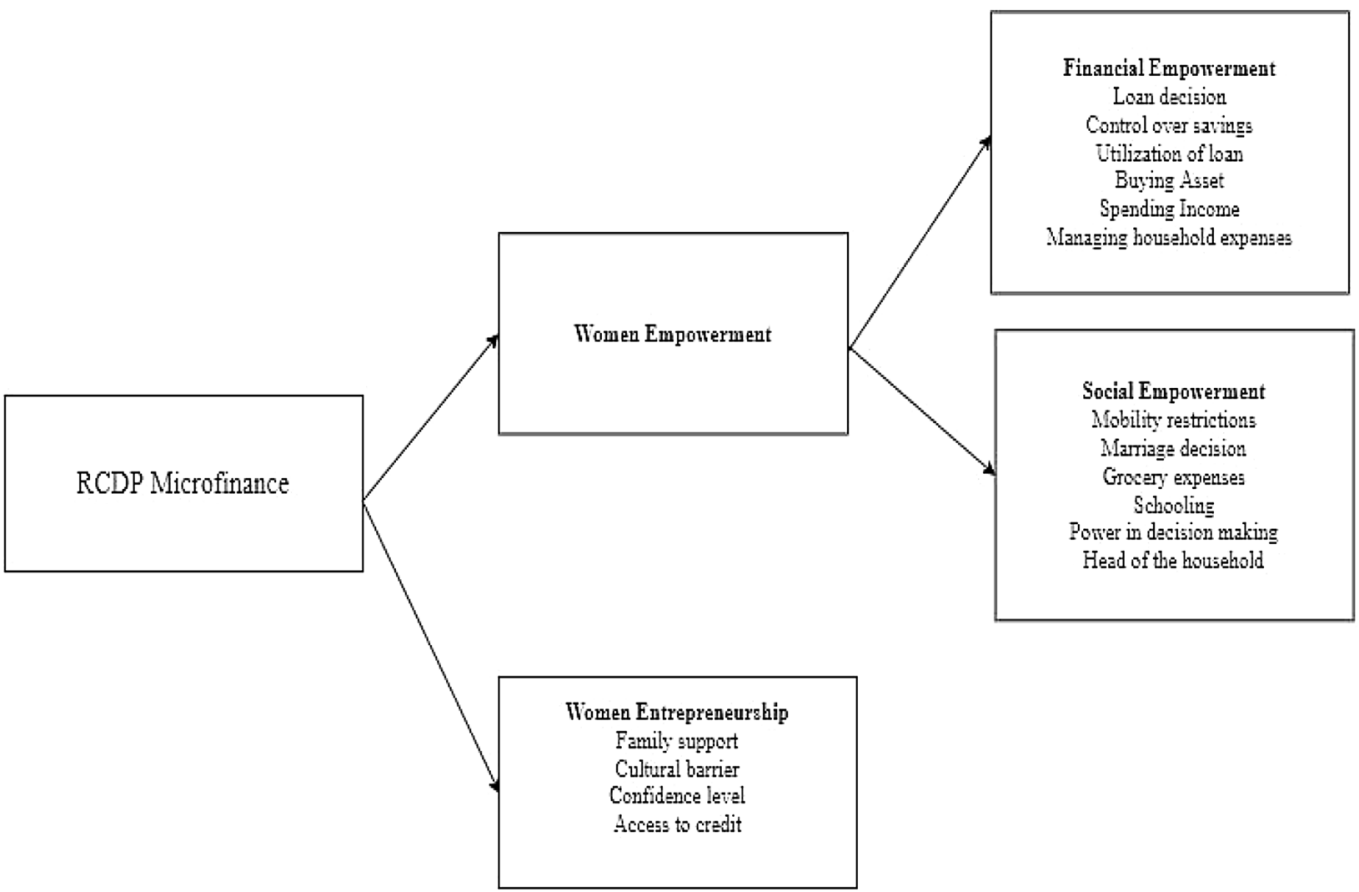
Conceptual framework of the study

The second theory used in this study is the status withdrawal theory, this theory explains that when certain groups of people realize that they are not respected by society. They switch to entrepreneurship for getting respect from society [22]. Thus, entrepreneurship is a function of status withdrawal. We follow this theoretical framework for understanding the entrepreneurial development among women borrowers. As all women borrowers belong to a poor class so we will explore whether they have any status withdrawal intention behind starting their own business or not (Fig. 1).

## Methodology

This study adopts the case study design approach for the empirical investigation as it inspects a contemporary phenomenon within the real life of participants, particularly when the limits between the context and phenomenon are not visible [59, 85]. The case study design is the most suitable design for this study to carefully understand the impact of MFIs on women’s empowerment as it provides more in-depth views about the phenomenon under study.

The variables and themes analyzed in the focus group discussions and in-depth interviews are presented in Table 1.

**Table 1:** Themes and variables of the study

Areas—in-depth interview and focus group	Variables
Demography	Education, age, gender, family size
Lifestyle	Occupation, income, micro-business
Empowerment	Factors leading to empowerment (social and financial)
Entrepreneurship	Access to seed money, control on loan utilization

### Semi-structured interviews

A qualitative method, in particular, semi-structured interviews, and a focus group were employed in this study. We used an interview checklist for the collection of qualitative data as it helps to properly understand the psychology of the participants. Also, it helped us to identify missing information from the participants. All interviews were conducted by telephone. The participants were selected through purposive sampling, as it is widely used in information-rich case studies [56]. The MFI selected for this study is RCDP, this MFI has played a key role in developing economic activities in communities and it exclusively focuses on women. The sample size consisted of six participants, who are aged 35 or above. Semi-structured interviews were organized in two sections, the *first section* included background questions based on the loan history of participants at the RCDP, demographic details, and a description of current business progress. The s*econd section* comprised questions that were related to participants’ viewpoints about their experience of gaining empowerment. For example, respondents were asked to provide in-depth explanations regarding their daily tasks and how their tasks get influenced after getting microloans. We also used sample prompts such as, “What role has microfinance played in your life?” and “Have you experienced any change due to microfinance? How it supported you in establishing your business?” Grand tour questions were also used such as “How would you explain a usual work week?” The grand tour questions lead us to get in-depth information through mini-tour questions for determining the details about certain events and the experience of women borrowers [69], such as Could you describe to me what you do for the mid-day meal when you are at your business? This helped in inquiring about delicate features such as advantages and changes associated with the role of microfinance in enhancing women’s empowerment. After conducting the semi-structured interviews, a focus group discussion with borrowers was conducted. This discussion helped us to collect data about the socioeconomic factors of women’s empowerment. This method helped us to have firsthand information (Table 2).

**Table 2:** Details of participants

Participant codes	Age	Marital status	Education	Business type	Number of dependents (children)	Monthly income (Rs)
Participant 1	35	Widow	Illiterate	Foodstuff retail	6	50,000
Participant 2	40	Divorced	Primary	Weaving and dressmaking	7	56,000
Participant 3	38	Married	Illiterate	Small bakery	3	61,000
Participant 4	36	Widow	Illiterate	Weaving and dressmaking	6	47,000
Participant 5	39	Married	Primary	Small Workshop	7	58,000
Participant 6	37	Married	Primary	Retail store	6	69,000

### Focus group

To analyze the experience and interactions among participants, a focus group plays an important role. Through focus groups, we probed answers to the best lending practice, saving plans, and effective interpersonal relationships between members. The group discussion helped us to make certain aspects clearer.

## Data analysis, results, and discussion

### Developing first-order codes and second-order themes

For analyzing the data, thematic analysis is used. First, to form codes, the data analysis started with coding iteratively, recorded interviews were used in performing the analysis [19]. At the initial stage, the data is linked with first-order codes that focus on the main research topic, the impact of MFIs on women’s empowerment. After this, common themes were used to join data fragments together from different but interconnected categories developed in the open coding [70]. This helped in combining first-order with second-order codes in a more precise manner.

### Incorporating first-order codes with second-order themes

In the second phase, the data was revisited to ensure precision in the second-order themes. The existing themes were refined or used to create new second-order themes. We analyzed the constructs for ensuring that the themes are reflecting first-order themes. For example, first-order coding statements related to respondents’ increased level of independence in decision making led us to form a second-order theme explaining “increase in independence in decision-making power.”

Later this statement was defined as “Social Empowerment” described by the first-order coding statements explaining independence to decide without asking anyone. This analysis adds precision in this phase, while simultaneously permitting us to better examine and improve other evolving concepts, such as “being independent.”

### Accumulating the theoretical dimensions

After second-order themes, we determined the theoretical dimensions for understanding the interaction among themes. For instance, some themes represented real experiences of social empowerment (e.g., “autonomy in decision making”) while others related to their response to social empowerment (e.g., “confidence in expressing an opinion”). We examined multiple models to check how multiple conceptual models relate to each other, using existing empowerment theory whenever suitable. We evaluated potential models against the data to investigate how emergent theoretical understanding described our research model. Table 3 presents the methodology, presenting the first-order codes, the second-order codes, and the theoretical dimensions that effectively describe the lived experiences of participants and the impact of microfinance in gaining empowerment.

**Table 3:** Overview of data structure

Descriptive codes	Second-order themes	Theoretical dimensions
•Can share my viewpoint in front of my family members •Can ask questions and disagree with the opinion of family members without any fear	Confidence in expressing an opinion	Social empowerment
•Can participate in household decisions •Can make decisions regarding asset purchase and loan utilization	Autonomy in decision making	
•Have ownership of valuable assets •Control over savings and cash	Control on assets	Financial empowerment
•Able to contribute to household income •Prepared for unexpected expenses	Control on household spending	
•Can manage daily food expenses •Can provide shelter to my children	Basic needs are managed	Improvement in lifestyle
•Can afford better health facilities •Able to purchase good clothes for my children	Improvement in living standards	
•Own desire to independently support a family •A powerful drive for earning income	Self-motivated	Entrepreneurial spirit
•Experienced several challenges in life since childhood •Able to face any unexpected challenge in life	Tolerant of ambiguity	
•Difficult to give proper time to family •Difficult to manage time for house chores	Difficulty in fulfilling family responsibilities	Difficulty in achieving work-life balance
•Cannot fully concentrate on business because of children •Faced more societal challenges in managing the business because I am a “woman”	Difficulty in managing business	

Table 4 reveals the data supporting each second-order theme by presenting that microfinance has proved very beneficial for all six participants. Our main research question was to determine how microfinance increases women’s empowerment. Thus, our results presented that microfinance drastically changed the perception of women borrowers about living an independent life and societal dynamics. Fulfilling the necessities is one of the primary issues of poor people and due to this, they have to earn for each day’s expenses. Further, because of having no savings to rely upon, the lines between households and businesses are often not so clear. All our respondents reveal that now they feel more confident and empowered as compared to their earlier condition. All participants shared that they spend their income on fulfilling their household expenses such as children’s schooling and utility bills. The findings of this study were obtained through thematic analysis which is useful in conducting identification analysis and pattern reporting within data [12]. This study aimed to determine how microfinance is an effective tool for women’s empowerment, and how microfinance leads to develop entrepreneurial characteristics among women, and how it is useful for women. The conclusions achieved from this study may not become generalizable for the whole population but it is generalizable at a conceptual level [30].

**Table 4:** Representative supporting data for each second-order theme

Second-order themes	First-order codes
(a) Confidence in expressing an opinion	After establishing and running my micro-business, I feel more confident in sharing my views and recommendations with my husband and in-laws Previously my husband had control over all family matters. But now I am also supporting my family by working with him so I don't feel any hesitation in suggesting any household matter and that makes me feel confident
(b) Autonomy in decision making	Earlier my husband was the sole decision maker. But now I participate equally in all household decisions. My in-laws also consider my suggestions regarding the utilization of loan amounts and other household decisions I take decisions related to savings, income, household expenses, and self-health care by myself
(c) Control over assets	I never owned any property in my life. But after getting a microloan, I purchased a small shop in my name I can keep all the cash and savings independently
(d) Control over household spending	Earlier my husband had control over all the savings and he used to make a household budget. He never saved money for unexpected expenses. But now I have control over household expenses and I make sure to save money to manage any unexpected challenge in life Being the sole earner now I manage and plan all household expenses. Being independent in spending decisions means happiness to me
(c) Basic needs are managed	My children and I slept many nights without having dinner. After starting my own business, now I can fully manage the basic needs of my children and shelter them I couldn’t believe that one day I will be able to fulfill the basic needs of my family
(d) Improvement in living standards	We have seen some very bad days in our lives. Microloans helped us to provide good clothes for our children I feel satisfied because now I can afford better health and education facilities for my children
(e) Self-motivated	I know that I am the only one who has to take care of my children. So, I stay motivated to earn for my family I am motivated because I know that only through my income, I can provide good education to my children
(f) Tolerant of ambiguity	I have gone through many difficult phases in life since my childhood. Now life has turned me obstinate I don’t have any fear to face any unexpected situation
(g) Difficulty in fulfilling family responsibilities	For doing business I have to spend the whole day at the shop. Due to this, I get very tired and I cannot cook for my children I feel difficulty in giving proper time to children and family
(h) Difficulty in managing business	When I am at work I kept on thinking about my children. I cannot fully concentrate on my business I have realized that running a business is more difficult for a woman in our society

The study determines the role of RCDP in women’s social and financial empowerment with the help of a case study methodology. We have used focus group discussions with in-depth interviews. We explored the lived experiences of women before and after taking a loan from RCDP and its impact on their social and financial empowerment with a view of William’s theory. In the focus group discussion, all participants shared their lived experiences and in the in-depth interviews, each case was analyzed for understanding the actual circumstances through which each participant has gone through. In this analysis, open-ended questions helped in understanding the real scenarios. The main research objective was to utilize open-ended questions for developing a comfortable association with the participants so that they can share all their lived experiences conveniently. We have selected in-depth interviews and focus groups because these methods were found more suitable for analyzing each case.

**Case 1** Participant (1) described when her husband died in a road accident. She became helpless. Her in-laws abandoned her with six children. Then, she applied for the microfinance program of RCDP and was provided with an initial loan worth Rs.75,000/- for establishing a small retail store of food items. In her village, no women were running their retail store. But she took this step to support her children and now she is running a successful business. The credit for her success goes to her decision of taking a loan and starting a new journey in her life. She expressed;

Life became miserable without my husband. It was difficult to feed six children. Without having a source of income and no place to stay. I felt that my life has come to an end. But microfinance helped me to get out of the crisis. Now I am living a peaceful life with my children.

**Case 2** Participant (2) shared that she remained in an abusive relationship with her husband for 11 years. Her husband was addicted to drugs. He divorced her after the birth of their seventh daughter. Then, he got married to some other woman. She expressed that she has gone through severe depression during that time when was alone with her daughters. Her mother and sister supported her but financially they were not capable to feed her children.

I was extremely depressed due to my divorce. I had no source of income other than him. I was worried about my daughters. I have four brothers and they also refused to support me at that time. Then, I started weaving and also started a dressmaking business on a small scale after taking a loan from RCDP. Particularly, I was good at making girls’ dresses. Now my mother and sister are living with me and I am supporting my family with my business.

**Case 3** Participant (3) expressed that microfinance helped her a lot in supporting her family. Initially, she took a loan of Rs 80,000 to start her business. She shared;

My husband was a plumber but his income was not enough to support the household expenses. Then a time came when my husband couldn’t find any job for three months. We were deprived of all necessities. And we also have three children who were not going to school due to our crisis. Then I asked my husband to start his own business of baking food items. Because I was good at baking. We both decided to take a loan and started our own business.

My husband was narrow-minded, initially, he refused to accept me as a partner in his business. But when he realized that only after one week our business showed visible growth. Then he allowed me to help him and we also hired two more workers. Now our children are going to school and we are managing all our household expenses.

**Case 4** Participant (4) expressed that her husband was an employee in a garment factory. One day the owner of the factory decided to wind up his business because of a lack of profits. My husband lost his job, he searched a lot for other jobs but he failed to find any suitable job. Then, he died due to a heart attack. She took a loan of Rs 60,000 for purchasing a sewing machine and some clothes. She shared

I was living a happy life with my husband and children but life changed when my husband lost his job. Further his death made the situation even worse. One of my neighbors told me about RCDP. Just because of my children I took a loan and started my own stitching business and now I am in a position to manage my all household expenses.

**Case 5** Participant (5) shared that her husband was employed in a workshop. But he lost his job due to the closure of that workshop. They had no other source of money. For four months, her husband searched for another job but he couldn’t find any opportunity. Their children left school because they were not able to pay their fees. Then, she convinced her husband to take a loan and start their own business. Her husband was afraid that we will not be able to repay the loan. Then, they will lose their respect in the family. But she told him that they have no other option and they have to take this risk.

My husband knew how to manage a car workshop so we decided to use the loan amount for starting a business. Gradually our business flourished, and we also managed to repay our monthly loan installments.

**Case 6** Participant (6) shared her life experiences by stating that her husband was an electrician and his income was not enough to support the family. They have six children and their school fees were not payable with that income. Thus, she asked her husband to start his own business as a retail store as there was no other store in their area.

I am managing a retail store with my husband. Initially, my husband took all decisions related to savings and asset purchases. But now as I am helping him in managing our retail store. He acknowledges my effort and now we collectively decide how to spend our income.

I and my husband started doing all chores together now. We both listen to each other, and collectively make decisions. He respects my suggestions and decisions. As we both couldn’t get a higher education, so we have realized the importance of education. Therefore, we are sending our children to good schools for quality education.

The credit for our success and better well-being goes not only to my hard work but to all including my family, friends, and also to RCDP who helped us to build up our lives once again.

We have found that after establishing their own business, women became more confident and self-empowered due to microfinance. They have developed a true belief in their entrepreneurial skills and independent decisions. These women are highly efficient as they not only make a business investment but also save some amount of money for future needs at the same time. Women use their amount of loans in smart investments in some entrepreneurial activities and in providing financial support to their families. But after becoming financially stable, they start saving money for future needs. This indicates the smart and strategic planning of women. After this phase, women are very confident in developing a strong position in their family and taking financial responsibility on their shoulders. These results also find support from past studies [53, 80]. Women have developed a serious working attitude toward their profession and are happy for supporting their husbands and family [77]. Hence, we can say that this all has become possible due to microfinancing as it not only provides financial support to women but also encourages them to contribute positively toward the development of society. [44]. Also, it plays a prominent role in establishing entrepreneurial knowledge and independent decision-making habit in women. Despite these efforts, many areas such as quality of services and working on new skill development trades, and gender responsiveness need improvement. The present form of this paper is not gender-friendly because it has mainly targeted the female gender and the male gender seems neglected. In addition to tangible development (food access and other necessities of life), it also provides intangible development to women in the form of motivation, self-belief, self-empowerment, confidence, and independent decision making. The findings of the study are in line with William’s theoretical model of women’s empowerment as the participants expressed that they have achieved empowerment by using their resources and agency. Further, the results are also in line with the status withdrawal theory as the participants expressed that they want to become independent because they want respect in society. Hence, our results are in line with the theories.

## Conclusion

Microfinance plays a dominant role to motivate and enhance entrepreneurial activities in any country. This study aims to examine the efficiency of microfinance in empowering women in Pakistan. The analysis and results revealed that microfinance is an effective tool that can contribute to the development of women’s empowerment and entrepreneurship. The findings also support the theoretical aspect of William’s theory as women empowerment is being discussed with a view of three dimensions including resources, agency, and achievements. The study contributed to breaking the conventional hurdles levied on women’s decisions and mobility. A developing country needs to focus on the growth and development of entrepreneurship for achieving stability. People find microfinance as an opportunity for themselves as it provides a way to enter into the entrepreneurship field. The six cases elaborated in this study reveal that the RCDP microfinance loan has been proven as a full-time and consistent earning source for the people and helped them a lot in improving their living standards. In the initial stage, the clients operated their business as sole proprietors, and over time, they involved many other people in the business. Thus, microfinance has become a potential source of earning for many needy people.

Hence, this study highlights that microfinance creates a positive and influential impact on rural women. It not only works for the betterment of women but also considers the entire families of those women by supporting them in enhancing their family earnings. In this way, this study will help in increasing the percentage of school-going children and a reduction in child labor due to an increase in family earnings. Although this project is concerned with providing small-scale services still it is contributing a lot toward the growth of Millennium Development Goals related to women’s empowerment, health, child welfare, and poverty alleviation. In light of these results, we came to know that microfinance has a diverse portfolio of benefits. It is not only a source of finance but also a tool that makes women more confident and boosts their morale. The findings indicate that even the small-scale loans taken from RCDP have helped women a lot to grow their socioeconomic and financial position through entrepreneurship which supports the theoretical foundation of the status withdrawal theory. It has benefited females with strong and independent decision-making power. Our results can help policymakers and practitioners to adopt suitable policies that assimilate empowerment in the formation of more effective projects for women. The findings of this study may encourage more women to take part in microfinance projects and entrepreneurial activities.

## Limitations and future directions

This study has some limitations. The first limitation is the shortage of time that resulted in designing a moderate sample size as compared to a bigger one. Second, the data collection is done for just one city and is limited to interest-based loans, whereas it has been found that RCDP is also concerned with interest-free loan programs. Third, the study used only six detailed interviews due to the time constraint factor which indicates that the findings cannot be fully generalized as only six cases were taken into account. However, this study has a potential scope in elaborating on all the possible dimensions of the related topic and it would enhance the recognition of women’s empowerment. This paper is dynamic as it covers both practical and theoretical aspects. Keeping in mind the time limitation and resource constraints, the above-discussed six cases can serve as a good starting step to allow the researcher to explore it further and investigate more dimensions in a longitudinal analysis. This study motivates women of our country to take a positive stand and contribute their role in poverty alleviation.

By documenting the limitations of the present study, future researchers are suggested to explore certain areas. Firstly, this study primarily deals with only a single project in the context of the most modern areas of Pakistan, it is recommended to future researchers perform this study in different regions of the country. Secondly, the sample size is limited due to time limitations; hence, future researchers are advised to conduct a study on the related topic by designing a large sample size. Lastly, it would be better for researchers to investigate the sustainability of community-based development projects via localized community-based adjustments with the support of local NGOs’ involvement rather than government funding.

## Data Availability

The data analyzed during the current study is available from the corresponding author on reasonable request.
